# Food Insecurity and Mental Health among Venezuelan Migrants and Refugees Living in Peru: Secondary Data Analysis of a Cross-Sectional Survey

**DOI:** 10.3390/nu15143102

**Published:** 2023-07-11

**Authors:** Akram Hernández-Vásquez, Fabriccio J. Visconti-Lopez, Alexandra C. Rojas-Cueva, Leandro Nicolás Grendas, Diego Azañedo

**Affiliations:** 1 Centro de Excelencia en Investigaciones Económicas y Sociales en Salud, Vicerrectorado de Investigación, Universidad San Ignacio de Loyola, Lima 15024, Peru; 2 Sociedad Científica de Estudiantes de Medicina—UPC, Universidad Peruana de Ciencias Aplicadas, Lima 15067, Peru; 3 Institute of Pharmacology, School of Medicine, University of Buenos Aires, Buenos Aires C1121ABG, Argentina; 4 Teodoro Alvarez Hospital, Buenos Aires C1406FWY, Argentina; 5 Faculty of Health Sciences, Universidad Científica del Sur, Lima 15067, Peru

**Keywords:** food insecurity, mental health, emigrants and immigrants, refugees, cross-sectional studies, Peru

## Abstract

The objective of this study was to analyze the association between food insecurity and mental health in Venezuelan migrants and refugees residing in Peru using data from the Survey Directed at the Venezuelan Population Residing in the Country (ENPOVE) conducted in 2022. The analysis included 7739 Venezuelan adults. The presence of mental health problems was self-reported, and household food insecurity was measured using the Food Insecurity Experience Scale. The study found that 4 out of 10 participants lived in households with moderate to severe food insecurity, and around 10% reported experiencing some mental health problem in the last month. The study identified a positive association between living in households with moderate to severe food insecurity and having some mental health problem compared to living in households without food insecurity. The findings suggest that food insecurity is a common problem among the Venezuelan migrant population residing in Peru, and measures are required to address this problem and mitigate its consequences on mental health and other health problems. The study highlights the need for international organizations to provide assistance and support to these populations and ensure adequate and sustainable follow-up of food insecurity at the national level. It is also necessary to implement early detection tests for mental health problems in the migrant population, especially in individuals exposed to food insecurity. This study provides relevant evidence for addressing public health in the Venezuelan migrant population residing in Peru.

## 1. Introduction

Venezuelan migration is one of the major humanitarian and social challenges that Peru has faced in recent years, being the second Latin American country, only after Colombia, with the largest number of Venezuelan migrants [1]. Over one million Venezuelan citizens, including children and adults, have arrived in the country in the last 10 years in search of better quality of life amidst the crisis and political instability in their country [1]. While migration has been beneficial in some aspects (such as increased labor productivity, gross domestic product growth, among others), it has also posed challenges for public health in the Peruvian context [2,3]. In particular, food insecurity is a frequent problem among the Venezuelan migrant population, who often face barriers to accessing adequate nutrition [4,5].

The lack of access to sufficient and nutritious food that enables an active and healthy life is known as food insecurity (FI) [6]. This public health problem is particularly common among people living in vulnerability and poverty worldwide, affecting more than 900 million people in 2021 [7]. In Peru, it is estimated that 60% of households of Venezuelan refugees and migrants were worried because they had insufficient food to eat due to lack of money or other resources in 2022 [8]. Similarly, in that same year, one in ten Venezuelan households reported skipping a full day of eating due to lack of resources [8]. In migrant households, causes of FI include lack of economic resources to acquire food and the absence of basic services (e.g., health and education) [9,10]. Relevant factors contributing to food insecurity among Venezuelan migrants in Peru are the lack of formal employment and job instability, which limit their income and capacity to purchase food, as well as the number of people living in the household [11]. Furthermore, it is important to acknowledge that there are several additional factors that have the potential to exert influence on this particular matter. Among these factors, one must consider the impact of migration trauma, which can encompass a range of psychological and emotional challenges experienced by individuals during the process of relocating from one place to another [12,13]. In addition to the aforementioned factors, it is crucial to recognize the substantial influence of sociocultural dynamics, which encompass an array of elements such as prevailing societal norms, deeply rooted values, and customary practices [14].

The association between food insecurity and mental health is a topic of increasing interest in scientific research. Various studies have documented that food insecurity can have adverse effects on people’s mental health [15,16]. Food insecurity can generate uncertainty about the ability to acquire sufficient food, causing stress and contributing to the development of symptoms of anxiety and depression [17,18]. Additionally, acquiring food in socially unacceptable ways can induce feelings of alienation, powerlessness, shame, and guilt, which have been related to depression [15,17,18,19]. This association has been investigated in different populations, including those living in rural and urban areas, as well as in developed and developing countries [20]. Although, the available literature on this association in migrants is limited [21], despite the fact that, unlike the non-migrant population, they are exposed to particular problems such as lack of legal status, unemployment, and low income, which can exacerbate food insecurity and, in turn, mental health problems [22].

The Survey Directed at the Venezuelan Population Residing in the Country (ENPOVE) is a survey carried out in Peru with which we can obtain information on food security and nutrition in this population [8]. Additionally, it provides information on the demographics, employment, and health of this population, including the presence of any mental health problems in the last month. The objective of this study was to analyze the association between food insecurity and mental health in Venezuelan migrants in Peru using data from the ENPOVE 2022. Studying this association in the population of Venezuelan migrants could generate relevant evidence for addressing public health in this population.

## 2. Materials and Methods

### 2.1. Design and Study Population

This study presents a secondary analysis of existing data from the ENPOVE 2022 survey, which was conducted for the second time in Peru to determine the demographic, social, economic, vulnerability, and protection characteristics of the Venezuelan refugee and migrant population residing in Peru. The study population comprised Venezuelan nationals residing in private and collective households in the cities of Tumbes, Piura, Chiclayo, Trujillo, Chimbote, Ica, Arequipa, Lima, and Callao. The sample selection was probabilistic, stratified, and independent in each study city, and the total sample size was 3680 households of Venezuelan refugees and migrants. Further details on the study design, procedures, data collection, and questionnaires can be found in the technical sheet and report of the ENPOVE 2022 [8,23].

**Figure 1:**
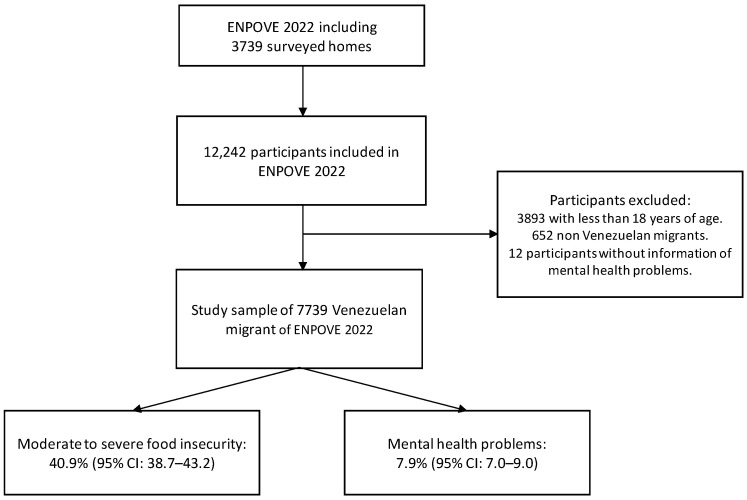
Flow chart of participants included in the study.

Of the 12,242 participants included in the ENPOVE 2022 survey, 8403 were adults (18 years or older). After excluding 652 non-Venezuelan participants and 12 who did not respond to the question on mental health problems, a total of 7739 participants were included in the final analysis (Figure 1).

### 2.2. Variables and Measurements

#### 2.2.1. Outcome Variable

The primary outcome variable in this study was the presence of mental health problems, which was measured through self-reporting by the participants. Although mental health problems can be screened using validated instruments confirmed by a psychiatrist, a self-reported response to the question, “In the last four weeks, have you had any discomfort or problem such as depression, fear, anger, anxiety, stress, etc.?” was used as a proxy variable for the presence of mental health problems. 

#### 2.2.2. Main Exposure

The main exposure variable in this study was household food insecurity, measured using the Food Insecurity Experience Scale (FIES) [24].

The ENPOVE 2022 included the following questions to measure food insecurity in any household member within the last 30 days: (1) Have you been very worried about not having enough food to eat? (WORRIED); (2) Were you able to eat healthy and nutritious foods or foods of your preference? (HEALTHY); (3) Did you eat only a few types of food? (FEWFOOD); (4) Did you skip a meal because of lack of food? (SKIPPED); (5) Did you eat less than you thought you should? (ATELLES); (6) Did your household run out of food? (RUNOUT); (7) Did you go hungry because you did not have enough money to buy food? (HUNGRY); and (8) Did you go without food for a whole day? (WHLDAY). All questions had Yes or No response options, which were recoded as 1 for affirmative responses and 0 for negative responses (for item 2, the assigned value was inverted), and then a score was obtained by summing all questions except for item 2. It should be noted that item 2 was excluded as it obtained a value higher than 1.5 in the infit statistic in the Rasch model performed using the RM weights package in R (version 4.2.1) (R Core Team, Vienna, Austria) [25,26]. Finally, a categorical variable was created based on the score obtained using the discrete assignment approach [27], which classified a household with moderate to severe food insecurity when they obtained a score of ≥4, mild food insecurity when they obtained a score between 1 and 3, and food security with a score of 0. This categorization has been used in previous studies [28,29].

#### 2.2.3. Covariables

The following variables were included as potential confounders considering that they were available in the ENPOVE 2022: sex (female, male), age group (18 to 29, 30 to 39, 40 to 49, 50 or more years), educational level that corresponds to the highest educational level attained (higher, secondary, up to primary), worked in the last week (no, yes), health insurance (no, yes), rented housing (no, yes), children under five years of age in the household (no, yes), older adults in the household (no, yes), household size (one person, 2 to 5, 6 or more people), and wealth index tercile (lower, middle, higher). The wealth index tercile was calculated based on a wealth index derived from a principal component analysis with variables on housing characteristics and household asset ownership. The details of the variables used and the categorization used for the construction of this variable can be reviewed in the Table 1.

### 2.3. Statistical Analysis

**Figure 2:**
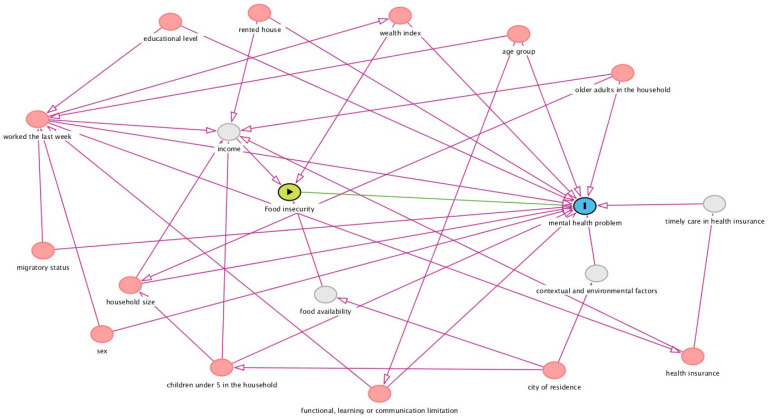
Directed Acyclic Graph (DAG) illustrating the causal associations between exposure, covariables, and outcome.

Scaled-weighted and sampling characteristics were used to account for the sample design of the ENPOVE 2022. Scaled-weighted characteristics were previously calculated following the scale-weighted method A described by Carle [30], in order to include a random effect at the household level, which takes into account the clustered nature of the data at this level. The study employed three types of analysis: univariate, bivariate, and multiple regression. The absolute frequency and weighted percentages of the variables were explored in the univariate analysis. Participant or household characteristics included in the study were compared with the exposure and outcome variable using bivariate analysis employing the chi-square test with Rao-Scott correction. Crude and adjusted prevalence ratios (PR) along with their 95% confidence intervals (CI) were calculated using a multilevel mixed-effects generalized linear model of the Poisson family. The chosen analysis approach accounted for the clustered nature of the data at the exposure level. To address potential confounding factors in the adjusted analysis, an epidemiological criterion was followed, and a Directed Acyclic Diagram (DAG) was constructed using DAGitty (Figure 2) [31]. A variable was identified as a confounder if it met the following three criteria: (1) it has an association with the exposure; (2) it has an association with the outcome; and (3) it is not on the causal pathway between the exposure and outcome [32]. The minimal sufficient adjustment set given for DAGitty was used in the multiple regression. Statistical analyses were performed using Stata v 17.0 (StataCorp, College Station, TX, USA). Statistical significance was set at a *p*-value < 0.05.

### 2.4. Ethical Considerations

The study did not require approval from an ethics committee as it involved aggregated secondary data that are publicly available and cannot identify the evaluated participants. The dataset is available for free download at https://proyectos.inei.gob.pe/microdatos/consulta.asp?cmbencuesta=ENCUESTA+DIRIGIDA+A+LA+POBLACI%D3N+VENEZOLANA+QUE+RESIDE+EN+EL+PA%CDS+-+ENPOVE&cmbanno=2022&cmbTrimestre=67 (accessed on 25 February 2023). All respondents provided informed consent to participate. All authors had access to the study data, and reviewed, and approved the final manuscript.

## 3. Results

Approximately half of the sample were women (51.6%), aged 18 to 29 years (42.6%), from the high wealth index tertile (35.3%), with secondary education (43.9%), worked the week previous to the survey (75.9%), had no health insurance (80.8%), and lived in a rented house (93.6%). Likewise, the majority of the respondents lived in households without children under 5 years old (63.3%), without elderly adults (88.8%), and composed of 2 to 5 persons (73.6%). The frequency of mental health problems was 7.9%, and the majority of participants lived in households that presented moderate to severe food insecurity (40.9%) (See Table 2).

According to statistically associated characteristics, the participants who reported the highest frequency of mental health problems were women (9.9%), those with higher educational levels (9.8%), those who did not have work in the week prior to the survey (9.4%), and those who experienced moderate to severe food insecurity (10.9%) (see Table 3).

Female participants (41.9%), belonging to the lower wealth index tertile (56.3%), with a primary education level (50.3%), who did not work the week before the survey (47.9%), did not have health insurance (42.9%), lived in households with children under 5 years old (45.8%), or without older adults (41.4%), lived in households with more than 6 people (49.1%), and who reported some mental health problem (56.2%), had the highest frequencies of moderate to severe food insecurity among the significantly associated characteristics (see Table 4).

In the simple regression analysis, a statistically significant association was identified between belonging to a household with moderate to severe food insecurity and having some mental health problem in the study population (PR: 2.05; 95% CI: 1.55–2.69). This association was strengthened after adjusting for sex, age group, wealth index tertile, health insurance, working status, rental housing, household size, household with a child under 5 years of age, and household with an older adult (PR: 2.16; 95% CI: 1.63–2.86) (see Table 5).

## 4. Discussion

The objective of this study was to analyze the association between food insecurity and mental health in Venezuelan migrants and refugees residing in Peru. It was found that 4 out of 10 participants lived in households with moderate to severe food insecurity, while around 10% reported having experienced some mental health problem in the last month. A positive association was identified between living in households with moderate to severe food insecurity and having some mental health problem, compared to those living in households without food insecurity. These findings suggest that food insecurity is a common problem among the Venezuelan migrant population residing in Peru, and that measures are required to address this problem and mitigate its consequences on mental health and other health problems that could affect the economy and efficiency of health care systems in the future.

The study revealed that nearly half of the participating Venezuelan migrants in Peru lived in households with moderate to severe food insecurity. Although this figure is higher than what has been reported in other studies of migrants in Portugal (10.7%) and Australia (13.7%) [9,33], even higher frequencies of food insecurity have been found in other populations, such as Haitian migrants in Chile (78%), Venezuelans in Trinidad and Tobago (86.6%), and refugees from Africa and the Middle East in high-income countries (40–71%) [34,35,36]. The differences in estimates of food insecurity between these populations may be due to the use of different measurement instruments, such as the Latin-American and Caribbean Food Security Scale [34], FIES at the individual level [35], and United States Department of Agriculture Household Food Security Survey [36], among others. Additionally, the study on Venezuelan migrants in Trinidad and Tobago [35] recruited participants during the COVID-19 pandemic, which may have increased the prevalence of food insecurity at that time. Furthermore, non-probabilistic recruitment methods, such as convenience and snowball sampling, used in some studies may affect the representativeness of the results and decrease their external validity [34,35,36]. On the other hand, the high prevalence of food insecurity in refugees living in high-income countries may be due to the difficulty that migrants face in finding formal jobs in these countries [36]. Moreover, the circumstances which cause people to migrate from their home countries, such as civil war, famine, poverty, and lack of opportunity, can significantly influence the results observed in these different groups. These underlying factors shape the experiences and vulnerabilities of individuals and families who embark on the journey of migration, ultimately impacting their access to resources and their ability to attain food security in their host countries. Despite the differences in results, it is evident that food insecurity in migrant and refugee populations is a global public health problem that has persisted over the years. Governments and non-governmental agencies play a crucial role in addressing the situation by implementing various measures. These measures should include enhancing access to nutritious food, strengthening social support systems, promoting cultural integration and social cohesion, and facilitating access to social benefits and entitlements. It is important to recognize that these actions are necessary within the context of sustainable development goals, ensuring a comprehensive and long-term approach to effectively tackle the challenges at hand [37].

The present study found that the frequency of mental health problems in the Venezuelan migrant population in Peru was 7.7%. Migrants and refugees are more prone to mental health disorders, such as post-traumatic stress disorder, anxiety, and depression, compared to those who reside in the host country [38,39]. This may be due to factors such as loneliness and isolation, which can affect the mental health of migrants and refugees [40,41] by hindering the expression and communication of their mental health problems. Additionally, the perception of not being accepted in the host country is related to higher rates of mood and anxiety disorders [42]. Experiences of discrimination and exclusion are also associated with elevated levels of emotional problems, stress, anxiety, and depression among migrants [43,44,45]. On the other hand, it is common for refugees to have to abandon most of their possessions, and as a result, many experience poverty, which can compromise their mental health. They often remain in this situation for years, regardless of their previous socioeconomic status [46,47,48]. While the social determinants of mental health that affect the general population are the same as those that influence the mental health of migrants and refugees, the nature of the migration experience, as well as social attitudes towards them, increase the likelihood of experiencing mental health problems after migration [49]. Therefore, it is necessary for the migrant population to have timely access to prevention, early detection, and adequate mental health treatment programs to improve their overall well-being and their ability to integrate into their new host country.

The probability of mental health problems among participants from households with moderate to severe food insecurity was 2.14 times higher than in those without food insecurity. These results are consistent with evidence described in various studies conducted in the general population, including a meta-analysis published in 2020 that included 19 studies from different continents (America, Europe, Africa, and Asia) and demonstrated a positive relationship between food insecurity and depression (odds ratio [OR] = 1.40; 95% CI: 1.30–1.58) and stress (OR = 1.34; 95% CI: 1.24–1.44) [21]. Additionally, a systematic review by Weaver and Hadley found a similar association in 16 studies conducted in different developing countries [50]. In the migrant population, an association has also been identified between food insecurity and depression (OR = 1.92; 95% CI: 1.33–2.78) and anxiety (OR = 1.93; 95% CI: 1.38–2.69). However, this study used only two questions from the Short Form of the 12-month Food Security Scale to determine food insecurity [51]. Currently, the Food and Agriculture Organization recommends using the FIES, which was used in our study, for measuring food insecurity internationally [24]. Despite these differences, the results of both studies are consistent, with estimates of association strength higher than those reported in the general population. These findings underscore the need to implement policies and programs that address food insecurity, especially in vulnerable populations, to prevent and treat mental health problems.

The design of this study has inherent limitations, one of which is the inability to establish a causal relationship due to its cross-sectional nature. However, we have been unable to identify longitudinal studies that evaluate this association in the migrant population, and this study is one of the first approaches to studying this problem. The personal and sensitive nature of this topic may have led participants to adjust their responses to meet current social standards or those of their reference group, which could have resulted in incomplete or biased responses. Additionally, there may have been the possibility of interviewer bias, through error or omission, in recording the respondents’ answers. Furthermore, although we adjusted our analysis for possible confounding factors identified in our DAG, other variables associated with food insecurity and mental health problems, such as household income, number of employed adults within each household, and access to social and food support programs, were not included, because they were not collected in the ENPOVE. Therefore, there is the possibility of unmeasured confounding. Furthermore, there is the possibility of memory bias influencing the results, as the presence of discomfort or problems (depression, fear, anger, anxiety, stress, among others) in the last four weeks was measured. While there are specific instruments for measuring each of these outcomes, which could avoid the possibility of outcome misclassification, this information is not currently available for this population. Likewise, the ENPOVE survey allows for a first approximation of the problem under study, with data representative of approximately 80% of the Venezuelan population residing nationally, and it can help health professionals and government institutions develop national strategies and policies. Moreover, it enables the advancement of new and longitudinal research, focused on solution-oriented approaches, to enhance our comprehension of this issue. For instance, the implementation of food programs, public dining rooms, and social support initiatives dedicated to addressing food insecurity can be established. Additionally, early detection tests for mental health problems should be implemented specifically for the migrant population, particularly those who are exposed to food insecurity. These measures aim to provide comprehensive support and assistance to individuals in need, ultimately contributing to improved well-being and outcomes within this vulnerable population.

## 5. Conclusions

In conclusion, this study highlights that Venezuelan migrants and refugees in Peru who belonged to households with moderate to severe food insecurity were more likely to experience mental health problems. It is essential that international and Peruvian government organizations that provide assistance and support to these populations ensure adequate and sustainable follow-up of food insecurity at the national level, establishing some measures. Firstly, it is crucial to strengthen food assistance programs, support local food production, improve income opportunities, provide nutrition education, and foster collaboration among stakeholders to address food insecurity comprehensively. Secondly, there is a need to increase access to affordable mental health services, raise awareness, promote community-based support networks, provide training for healthcare professionals, and integrate mental health into primary healthcare settings to effectively address the mental health concerns of migrant and refugee populations.

## Figures and Tables

**Table 1:** Variables used for the construction of wealth index.

Code	Question	Answer	Categorization
P110_1	Does your household have: Color TV?	1 Yes	1 Yes
		2 No	0 No
P110_2	Does your household have: Gas stove?	1 Yes	1 Yes
		2 No	0 No
P110_3	Does your household have: Blender?	1 Yes	1 Yes
		2 No	0 No
P110_4	Does your household have: Electric iron?	1 Yes	1 Yes
		2 No	0 No
P110_5	Does your household have: Computer/laptop/tablet?	1 Yes	1 Yes
		2 No	0 No
P110_7	Does your household have: Landline phone?	1 Yes	1 Yes
		2 No	0 No
P110_8	Does your household have: Radio?	1 Yes	1 Yes
		2 No	0 No
P110_9	Does your household have: Refrigerator/freezer?	1 Yes	1 Yes
		2 No	0 No
P110_10	Does your household have: Washing machine?	1 Yes	1 Yes
		2 No	0 No
P108_1	Does the water you use at home come mainly from:	Public network, inside the house?	1 Yes
		Public network, outside the house, but inside the building?	0 No
		Pilon or pool for public use?	0 No
		Truck-tanker or other similar?	0 No
		Other?	0 No
P108_2	Is the bathroom or toilet in your home connected to:	Public drainage network inside the house?	1 Yes
		Public drainage network outside the home, but inside the building?	0 No
		Latrine (with treatment)?	0 No
		Septic tank, septic tank or biodigester?	0 No
		Other?	0 No
P108_4	Does your home have internet service?	1 Yes	1 Yes
		2 No	0 No
P102	Is the predominant construction material for exterior walls:	Brick or cement block?	1 Yes
		Stone, ashlar with lime or cement?	0 No
		Adobe?	0 No
		Rammed earth?	0 No
		Quincha (cane with mud)?	0 No
		Stone with mud?	0 No
		Wood (pona, screw, etc.)?	0 No
		Plywood/calamine/mat?	0 No
		Other material?	0 No
P103	Is the predominant construction material in the roofs:	Reinforced concrete?	1 Yes
		Wood?	0 No
		Roof tiles?	0 No
		Calamine sheets, fiber cement or similar?	0 No
		Cane or mat with cake of mud or cement?	0 No
		Plywood/mat/reed?	0 No
		Straw, palm fronds, etc.?	0 No
		Other material?	0 No
P104	Is the predominant material in the floors:	Parquet or polished wood?	1 Yes
		Asphalt sheets, vinyl or similar?	1 Yes
		Tiles, terraces or similar?	1 Yes
		Wood (pona, screw, etc.)?	0 No
		Cement?	0 No
		Land?	0 No
		Other material?	0 No
P105	How many rooms in total does the house have, without counting the bathroom, kitchen, hallways, and garage?	Number of rooms	1 More than one inhabitant per room 0 One or less than one inhabitant per room
P15	Does anyone who came from Venezuela live in this home? (at least one person of Venezuelan nationality)	1 Yes	
		2 No	
P15_N	Number of people	Number of people	

**Table 2:** Characteristics of the population of adult Venezuelan migrants residing in Peru participating in ENPOVE 2022 included in the study (*n* = 7739).

Characteristics		*n*	% (95% CI)
Sex			
	Female	3999	51.6 (50.7–52.6)
	Male	3740	48.4 (47.4–49.3)
Age group			
	18 to 29 years	3286	42.6 (41.1–44.0)
	30 to 39 years	2408	31.4 (30.1–32.7)
	40 to 49 years	1124	14.6 (13.6–15.5)
	50 to more	921	11.5 (10.7–12.4)
Wealth index tertile			
	Lower	2408	30.6 (28.6–32.7)
	Middle	2668	34.0 (32.0–36.1)
	Higher	2663	35.3 (33.1–37.6)
Educational level			
	Higher	3265	43.1 (41.5–44.8)
	Secondary	3451	43.9 (42.3–45.7)
	Up to primary	1023	12.9 (11.7–14.2)
Worked the last week			
	No	1900	24.1 (23.1–25.2)
	Yes	5839	75.9 (74.8–76.9)
Health Insurance			
	No	6293	80.8 (79.4–82.1)
	Yes	1446	19.2 (17.9–20.7)
Rented house			
	No	476	6.4 (5.5–7.5)
	Yes	7263	93.6 (92.5–94.5)
Children under 5 in the household			
	No	4931	63.3 (61.3–65.4)
	Yes	2808	36.7 (34.6–38.7)
Older adults in the household			
	No	6838	88.8 (87.2–90.2)
	Yes	901	11.2 (9.8–12.8)
Household size			
	1 person	697	8.9 (8.1–9.7)
	2 to 5 persons	5754	73.6 (71.5–75.6)
	6 or more persons	1288	17.5 (15.5–19.7)
Mental health problem			
	No	7140	92.0 (90.9–92.9)
	Yes	599	7.9 (7.0–9.0)
Household food insecurity			
	Food security	1878	24.3 (22.4–26.3)
	Mild	2721	34.8 (32.8–36.9)
	Moderate to severe	3140	40.9 (38.7–43.2)

Estimates include the weights and ENPOVE 2022 sample specifications. CI: confidence interval.

**Table 3:** Characteristics associated with mental health problems in the last month in the population of adult Venezuelan migrants residing in Peru participating in ENPOVE 2022 included in the study (*n* = 7739).

Characteristics		Mental Health Problem in the Last Month		*p*-Value
		No (*n* = 7140) *n* (%)	Yes (*n* = 599) *n* (%)	
Sex				<0.001
	Female	3617 (90.1)	382 (9.9)	
	Male	3523 (94.1)	217 (5.9)	
Age group				0.079
	18 to 29 years	3028 (92.3)	258 (7.7)	
	30 to 39 years	2237 (92.9)	171 (7.1)	
	40 to 49 years	1022 (89.8)	102 (10.2)	
	50 to more	853 (91.4)	68 (8.6)	
Wealth index tertile				0.496
	Lower	2230 (92.6)	178 (7.4)	
	Middle	2443 (91.4)	225 (8.7)	
	Higher	2467 (92.1)	196 (7.9)	
Educational level				<0.001
	Higher	2956 (90.2)	309 (9.8)	
	Secondary	3226 (93.4)	225 (6.6)	
	Up to primary	958 (93.3)	65 (6.7)	
Worked the last week				0.018
	No	1723 (90.6)	177 (9.4)	
	Yes	5417 (92.5)	422 (7.5)	
Health Insurance				
	No	5803 (91.9)	490 (8.1)	0.414
	Yes	1337 (92.7)	109 (7.3)	
Rented house				0.074
	No	446 (94.4)	30 (5.6)	
	Yes	6694 (91.9)	569 (8.1)	
Children under 5 in the household				0.87
	No	4559 (92.1)	372 (7.9)	
	Yes	2581 (91.9)	227 (8.1)	
Older adults in the household				0.259
	No	6315 (92.3)	523 (7.8)	
	Yes	825 (90.2)	76 (9.8)	
Household size				0.187
	1 person	628 (89.7)	69 (10.3)	
	2 to 5 persons	5321 (92.1)	433 (7.9)	
	6 or more persons	1191 (92.7)	97 (7.3)	
Household food insecurity				<0.001
	Food security	1782 (94.8)	96 (5.3)	
	Mild	2549 (93.6)	172 (6.4)	
	Moderate to severe	2809 (89.0)	331 (10.9)	

Estimates include the weights and ENPOVE 2022 sample specifications.

**Table 4:** Characteristics associated with household food insecurity in the last month in the population of adult Venezuelan migrants residing in Peru participating in ENPOVE 2022 included in the study (*n* = 7739).

Characteristics		Food Insecurity in the Last Month			*p*-Value
		No (*n* = 1878) *n* (%)	Mild (*n* = 2721) *n* (%)	Moderate to Severe (*n* = 3140) *n* (%)	
Sex					0.005
	Female	924 (23.1)	1413 (34.9)	1662 (41.9)	
	Male	954 (25.5)	1308 (34.7)	1478 (39.8)	
Age group					0.379
	18 to 29 years	811 (25.1)	1133 (33.6)	1342 (41.3)	
	30 to 39 years	561 (23.4)	867 (35.7)	980 (40.9)	
	40 to 49 years	281 (25.1)	382 (33.7)	461 (41.2)	
	50 to more	225 (22.4)	339 (38.2)	357 (39.4)	
Wealth index tertile					<0.001
	Lower	372 (15.7)	699 (28.0)	1337 (56.3)	
	Middle	555 (20.9)	1014 (37.3)	1099 (41.8)	
	Higher	951 (34.9)	1008 (38.3)	704 (26.8)	
Educational level					<0.001
	Higher	944 (29.7)	1177 (35.5)	1144 (34.8)	
	Secondary	772 (21.7)	1187 (34.1)	1492 (44.2)	
	Up to primary	162 (14.9)	357 (34.7)	504 (50.3)	
Worked the last week					<0.001
	No	373 (19.3)	621 (32.7)	906 (47.9)	
	Yes	1505 (25.9)	2100 (35.5)	2234 (38.7)	
Health Insurance					<0.001
	No	1403 (21.9)	2232 (35.1)	2658 (42.9)	
	Yes	475 (34.4)	489 (33.4)	482 (32.3)	
Rented house					0.098
	No	139 (28.8)	171 (38.1)	166 (33.0)	
	Yes	1739 (23.9)	2550 (34.6)	2974 (41.5)	
Children under 5 in the household					<0.001
	No	1349 (27.6)	1717 (34.3)	1865 (38.1)	
	Yes	529 (18.6)	1004 (35.6)	1275 (45.8)	
Older adults in the household					0.048
	No	1681 (24.7)	2357 (33.8)	2800 (41.4)	
	Yes	197 (20.7)	364 (42.3)	340 (37.0)	
Household size					<0.001
	1 person	244 (36.6)	203 (28.5)	250 (34.9)	
	2 to 5 persons	1405 (24.6)	2072 (35.7)	2277 (39.7)	
	6 or more persons	229 (16.7)	446 (34.2)	613 (49.1)	
Mental health problem					<0.001
	No	1782 (25.0)	2549 (35.4)	2809 (39.6)	
	Yes	96 (15.9)	172 (27.8)	331 (56.2)	

Estimates include the weights and ENPOVE 2022 sample specifications.

**Table 5:** Association of household food insecurity during the last month and having any mental health problem in the population of adult Venezuelan migrants residing in Peru participating in ENPOVE 2022 included in the study (*n* = 7739).

Characteristics		Bivariate Analysis			Multiple Regression *		
		PR	95% CI	*p*-Value	PR	95% CI	*p*-Value
Household food insecurity							
	Food security	Ref.			Ref.		
	Mild	1.18	0.88–1.57	0.259	1.22	0.92–1.63	0.169
	Moderate to severe	2.05	1.55–2.69	<0.001	2.16	1.63–2.86	<0.001

* Adjusted for sex, age group, wealth index tertiles, health insurance, working status, rental housing, household size, household with a child under 5 years of age, household with an older adult. Estimates include the weights and ENPOVE 2022 sample specifications. PR: Prevalence ratio. CI: confidence interval.

## Data Availability

The dataset is available for free download at https://proyectos.inei.gob.pe/microdatos/consulta.asp?cmbencuesta=ENCUESTA+DIRIGIDA+A+LA+POBLACI%D3N+VENEZOLANA+QUE+RESIDE+EN+EL+PA%CDS+-+ENPOVE&cmbanno=2022&cmbTrimestre=67 (accessed on 25 February 2023).
